# Cross-Sectional Seroepidemiologic Study of Coronavirus Disease 2019 (COVID-19) among Close Contacts, Children, and Migrant Workers in Shanghai

**DOI:** 10.3390/ijerph17197223

**Published:** 2020-10-02

**Authors:** Shuang-Fei Xu, Yi-Han Lu, Tao Zhang, Hai-Yan Xiong, Wei-Bing Wang

**Affiliations:** Department of Epidemiology, School of Public Health, Fudan University, Shanghai 200032, China; 19111020007@fudan.edu.cn (S.-F.X.); luyihan@fudan.edu.cn (Y.-H.L.); tzhang@shmu.edu.cn (T.Z.); haiyanxiong@fudan.edu.cn (H.-Y.X.)

**Keywords:** seroepidemiologic study, Coronavirus Disease 2019 (COVID-19), Shanghai

## Abstract

(1) Background: Along with an increasing risk caused by migrant workers returning to the urban areas for the resumption of work and production and growing epidemiological evidence of possible transmission during the incubation period, a study of Coronavirus Disease 2019 (COVID-19) is warranted among key populations to determine the serum antibody against the SARS-CoV-2 and the carrying status of SARS-CoV-2 to identify potential asymptomatic infection and to explore the risk factors. (2) Method: This is a cross-sectional seroepidemiologic study. Three categories of targeted populations (close contacts, migrant workers who return to urban areas for work, and school children) will be included in this study as they are important for case identification in communities. A multi-stage sampling method will be employed to acquire an adequate sample size. Assessments that include questionnaires and blood, nasopharyngeal specimens, and feces collection will be performed via home-visit survey. (3) Ethics and Dissemination: The study was approved by the Institute Review Board of School of Public Health, Fudan University (IRB#2020-04-0818). Before data collection, written informed consent will be obtained from all participants. The manuscripts from this work will be submitted for publication in quality peer-reviewed journals and presented at national or international conferences.

## 1. Introduction

Since the first known case of pneumonia infected with the novel coronavirus was reported in the city of Wuhan in late December of 2019, Coronavirus Disease 2019 (COVID-19), caused by SARS-CoV-2 and announced by the World Health Organization on 11 February 2020, unexpectedly and quickly spread in China and many other countries with rapid geographical expansion and a sudden increase in the number of cases [1,2]. On January 20, COVID-19 was added into China’s “Law on Prevention and Control of Infectious Diseases” as a Class B notifiable disease and necessitated prevention and control measures as a Class A because of its emergences [3]. On 23 January, the Chinese Government began to limit population movement in and out of Wuhan. In the following week, provinces in mainland China successively launched Response Level I of the Major Public Health Emergency to respond to the COVID-19 epidemic, including the lockdown of whole cities, cancelation of celebration activities of Spring Festival (Chinese New Year), and deferral of attendance at school and work.

To our knowledge, the common clinical features of COVID-19 are non-specific, such as fever, dry cough, and bilateral and peripheral ground-glass and consolidative pulmonary opacities on chest computed tomography (CT) scans, in addition to other symptoms including dyspnea, headache, muscle soreness, and fatigue [4,5]. According to the released data from the Chinese Center for Disease Control and Prevention, the overall case-fatality rate (CFR) was 2.3%, though the CFR was much higher among the critical (49%) and the elderly cases (20–30%). Of the total cumulative confirmed cases on February 11, 2020, the majority (81%) were classified as non-pneumonia and mild pneumonia, and no death has been documented among those with mild or severe symptoms [6]. At the end of February 2020, a total of 79,824 confirmed COVID-19 cases were recorded in mainland China, of whom 41,625 had been discharged and 2870 had died [7]. Thus far, the majority of published studies focused on hospitals and confirmed/suspected cases rather than on the population at risk in communities. Along with the resumption of work and production activities and the adjustment of the emergency response level, an increasing number of migrant workers across the whole country are gradually returning to urban areas, which adds an extra burden on disease prevention and management. Meanwhile, several studies provided epidemiological evidence of possible transmission of SARS-CoV-2 from pre-symptomatic and asymptomatic cases (asymptomatic people with SARS-CoV-2 detected in respiratory specimens or immunoglobulin M (IgM) detected in serum) [8,9]. Thus, a cross-sectional seroepidemiologic study of COVID-19 among key populations is warranted to determine the potential risk of SARS-CoV-2 infection in different scenarios.

## 2. Materials and Methods

### 2.1. Ethical Approval

The study introduces no significant risk to participants, with no medication or intervention involved. The study was approved by the Institute Review Board of School of Public Health, Fudan University (IRB#2020-04-0818). Before data collection, the purpose and procedures of the study will be explained to all the eligible participants. Written informed consent will be obtained from all participants and from the parents of minors. Participants can withdraw from the study at any point without any adverse consequences. All data are anonymous and will be managed confidentially.

### 2.2. Study Objectives

To determine the serum antibody level against the SARS-CoV-2 and the carrying status of SARS-CoV-2 among key susceptible populations to identify potential asymptomatic infection and to explore the risk factors.

### 2.3. Study Population

Three categories of study participants will be recruited in the study as they are important for case finding in communities. Participants must meet the following inclusion criteria:

1. Close contacts: The definition of “close contacts” is based on *Prevention and Control of Novel Coronavirus Pneumonia (6th edition)*, which refers to people who had unprotected close contact (within 1 meter) with a confirmed or suspect case within two days before illness onset, or with an asymptomatic infected person within two days before sampling [10]. In China, the tracing and management of close contacts is implemented by the local Center for Disease Control and Prevention (CDC), and all identified close contacts are recorded in health administration departments.
  - Volunteer to participate in the survey and provide written informed consent.
2. Domestic migrant workers returning to urban areas for work:
  - Aged 18 years and above;
  - Unconfirmed COVID-19 cases;
  - Volunteer to participate in the survey and provide signed informed consent.
3. School children:
  - Aged above 6 years;
  - Attending primary school, middle school, or high school (non-vocational high school);
  - Unconfirmed COVID-19 cases;
  - Volunteer to participate in the survey and provide signed informed consent themselves and/or through their parent(s).
4. Exclusion Criteria
  - History of any neurologic disorders;
  - Language disorders.

### 2.4. Study Design

This is a cross-sectional study design. 

### 2.5. Sample Size

The sample size is calculated according to the following formula:

(1) $$n_{SRS}=\frac{z_{\alpha /2}^{2}p\left(1-p\right)}{d^{2}}$$

(2) $$n=n_{SRS}/\left(1-k\right)$$

where *nSRS* is the sample size under simple random sample assumption; *Zα*/2 is the statistic corresponding to level of confidence, assumed to be 1.96 (when *α* = 0.05); *d* is precision, assumed to be 20%*p [11]; *k* is the missing rate, assumed to be 10% [11]; n is the minimum required sample size; *p* is the expected seroprevalence of antibodies against SARS-CoV-2 among the target populations. However, these parameters for the above three categories of study participants remain unclear. Although the spread of SARS-CoV-2 is much faster than that of the SARS-CoV in 2003, these two coronaviruses share a similar transmission mode, such as airborne transmission and close person-to-person contact, via respiratory droplets from sneezing or coughing, and fomites. Thus, we consider referring to the transmission data of SARS epidemic. In 2003, after the SARS epidemic, the seroprevalence of antibodies against SARS-CoV tested by enzyme-linked immunosorbent assay (ELISA) among close contacts, general population and school children were 0.19–4.87%, 0.0083–2.26% and 0–1.70%, respectively [12,13,14,15,16]. Similarly, we assume the expected seroprevalence of SARS-CoV-2 among close contacts, migrant workers, and school children to be 5%, 3%, and 2%, respectively. If the research designers in different regions obtain more specific local data, they can adjust the calculations.

Finally, in our design, the expected sample sizes for close contacts, migrant workers, and school children are 2028, 3450, and 5228, respectively.

### 2.6. Sampling Strategies and Study Sites

Multi-stage sampling methods will be employed to acquire adequate sample size. Primary sampling units (PSUs) are sampled with a probability proportional to size (PPS), that is, the number of subunits within each PSU. Given the different population sizes of the three categories of study participants and the different sampling strategies, the selected PSUs for each target population may not be identical.

For close contacts, the specific sample size will be determined by the cumulative number of contacts in the city. It is best to include all contacts of the confirmed COVID-19 cases, to maximize the statistical power of the study. Otherwise, one-stage design with cluster sampling is chosen. Here, “size” in PPS refers to the number of close contacts in each district, which is now replaced by the size of confirmed COVID-19 cases because there are no open data about close contacts in Shanghai. The four districts, from the total 16, selected as PSUs with PPS sampling were Pudong district, Xuhui district, Yangpu district, and Songjiang district (Table 1). All eligible close contacts in the selected districts will be enrolled.

For migrant workers returning to the city for work, a two-stage design with PPS and successive sampling is chosen. At the first stage, PPS (“size” here refers to the floating population count in each district) sampling is employed to select four PSUs. Considering the distribution of floating population in each district released by the Shanghai Statistics Bureau in 2018, Pudong district, Putuo district, Baoshan district, and Qingpu district were selected (Table 2). At the second stage, a fixed number of individuals will be enrolled using successive sampling. For migrant workers, to our knowledge, migrants returning to Shanghai are required to actively register with the village/neighborhood committees since January, 2020. Thus, all eligible migrant workers in each PSU will be recruited one-by-one from the registers until the expected sample size is reached.

For school children, a three-stage design with PPS, simple random sampling (SRS), and cluster sampling is chosen. At the first stage, PPS (“size” here refers to the number of schools in each district at the second stage) sampling is employed to select 4 PSUs. Taking Shanghai for example again, based on the data from Shanghai Education Bureau [17], Pudong district, Jing’an district, Minhang district, and Songjiang district were selected (Table 3). At the second stage, schools in each district will be stratified as primary school, middle school, and high school, and SRS will be used to select 1~2 schools from each stratum. At the third stage, 1 or 2 classes in each grade will be chosen at random, and all eligible students should be enrolled (the specific number of classes can be adjusted by the admission size). The sample size for school children is “deff” (the design effect in cluster sampling, assumed to be 1.5), which multiplies the expected value (*n* = 5228).

### 2.7. Questionnaire

1. Sociodemographic characteristics: name, telephone (mobile) number, date of birth, sex, e-mail address, current address, ethnicity, job, educational level, parent employment status, educational level (only for children) and preferred mode of contact (telephone, email, or express delivery);
2. Underlying conditions: pregnancy, obesity, cancer, diabetes, hypertension, heart disease, asthma requiring medication, chronic lung disease (non-asthma), chronic liver disease, chronic hematological disorder, chronic kidney disease, chronic neurological impairment/disease, and other underlying conditions. In addition, respiratory-pathogen-related vaccinations will be reviewed.
3. Clinical symptoms within the last 14 days: body temperature, fever, chill, dry cough, sore throat, runny nose, shortness of breath, nausea, vomiting, diarrhea, and other symptoms.
4. General exposure information: possible contact with confirmed/suspected cases, visits to medical facilities, and travel history (including destination, transfer, and duration) within the last 14 days.

### 2.8. Laboratory Evaluation

1. Specimen collection, transportation, and storage

Appropriate personal protective equipment should be worn when specimens are being collected. Specimens of close contacts will be collected by the designated local CDCs and medical facilities. Specimens of migrant workers and school children will be collected by qualified technicians. All specimen containers should be labeled with the full name of the person being sampled, time and date of collection, and one other unique identifier such as the National Medical Insurance Number.

- Blood specimen: A 5 mL whole-blood sample will be collected with a vacutainer with no anticoagulant. Once the blood is drawn, the vacutainer should be inverted 5 or 6 times and placed at room temperature. When the blood specimen is sent to the laboratory, the vacutainer will be centrifuged for 10 min at 1500–2000 rpm at room temperature. Serum will be extracted by pipette and stored in a sterile spiral plastic tube.

In addition, another 5 mL whole-blood sample will be collected with a vacutainer containing EDTA anticoagulant. Once the blood is drawn, the vacutainer should be inverted at least 10 times and placed at room temperature for 30 min. Then the vacutainer will be centrifuged for 10 min at 1500–2000 rpm at room temperature. Plasma and blood cells will be separately collected into sterile spiral plastic tubes.

- Nasopharyngeal (NP) swab: Two NP swabs will be collected for each eligible participant. The swab will be directly put in the nose parallel to the base of the NP passage. The swab should move without resistance until reaching the nasopharynx, located about one-half to two-thirds the distance from the nostril to an ear lobe. If resistance occurs, the swab will be removed, and an attempt will be made to take the sample entering through the same or the other nostril. Once the swab reaches nasopharynx, the swab will be rotated 180°, or left in place for 5 s to saturate the swab tip; and then the swab will be removed slowly. Then the swab head will be inserted into the tube containing 3.5 mL of virus preservation buffer (Virus Transport Medium (containing Hank’s balanced salt solution, polymyxin B, vancomycin, bovine serum albumin, cryoprotectant, biobuffer, etc.), Shanghai Comagal Microbial technology CO. LTD.) and swab shaft will be evenly broken at the scored line to fit in tube and replace cap tightly.
- Feces or anal swab: 3–5 mL of stool that has not been mixed with urine will be collected in a clean, dry, leak-proof container. If it is not convenient to collect fecal samples, an anal swab can be collected. The disinfectant cotton swab will be gently inserted into the anus to a depth of 3–5 cm, then it will be gently rotated pulled out, and immediately put into a 15 mL screw-capped sampling tube containing 3–5 mL virus preservation buffer. Then, the swab shaft will be evenly broken at the scored line to fit in tube and the cap will be replaced tightly.

Blood specimens, NP swabs, and anal swabs should be taken at the home visit. Feces could be collected the next day. All specimens will be shipped to the laboratory in a sealed biohazard bag within 24 h after collection at 4 °C on ice packs. If transportation will be delayed more than 24 h, specimens should be reserved at −70 °C and shipped on dry ice. It is important to avoid repeated freezing and thawing of specimens.

2. Laboratory examinations
  - Serological testing: The serum specimen will be available for qualitative detection of SARS-CoV-2-specific total antibodies (including IgM, IgG, IgA, and other antibody types) with Novel Coronavirus (2019-nCoV) Antibody Test Kit (chemiluminescence immunoassay method) (registered number: 20203400198), developed by Xiamen InnoDx Biotech Co., Ltd. (Xiamen, China) and which is the world’s first approved total antibody detection reagent with the double-antigen sandwich method for SARS-CoV-2. It can rapidly and simply detect specific antibodies within 29 min.
  - Etiological testing: A real-time fluorescence-based reverse transcriptase-polymerase chain reaction (RT-PCR) assay will be applied to the NP specimen and feces to detect SARS-CoV-2. The primers and probes (targeting open reading frame 1ab (ORF 1ab) and nucleocapsid protein (N) in the novel coronavirus genome) used for SARS-CoV-2 detection by RT-PCR is from the *Novel Coronavirus Pneumonia: Laboratory Testing Guideline*, released by National Health Commission of the PRC. In addition, NP specimens will be further examined for a total of 41 respiratory pathogens (Table 4) via gene chips (Micro-Fluid Chip for Respiratory Pathogens, product number: 4398986).

In this cross-sectional survey, participants with serological evidence (SARS-CoV-2-specific IgM and IgG detectable in serum) or etiological evidence (real-time fluorescent RT-PCR indicating positive for SARS-CoV-2 nucleic acid) will be diagnosed as confirmed cases.

### 2.9. Study Procedure

**Figure 1:**
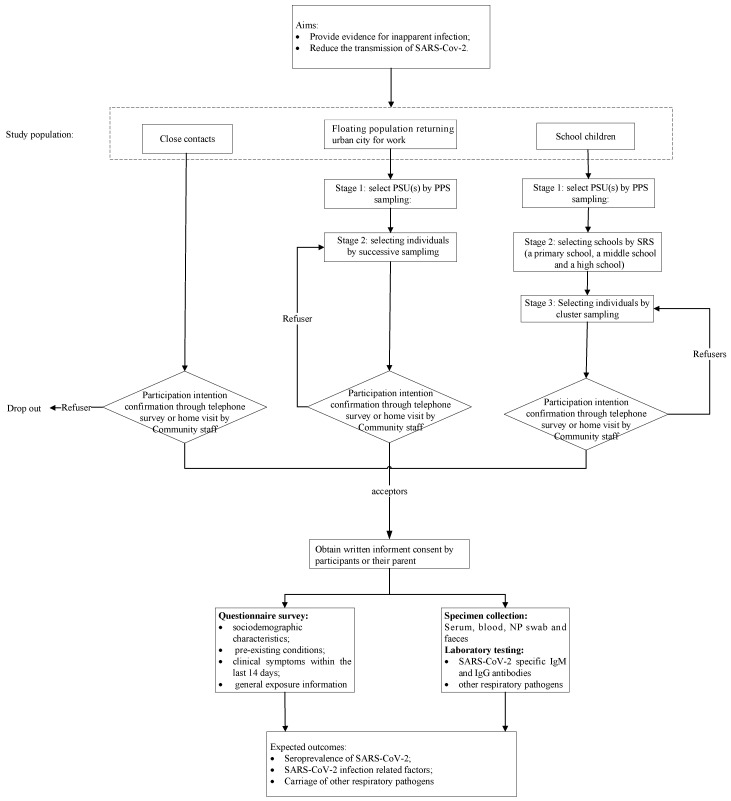
Study recruitment and data collection flow chart.

The study design is presented in Figure 1. Investigators will communicate with the selected participants or guardians in advance to assure their intention of participating this program. The refusers will be replaced by resampling without replacement in each PSU. Investigators will visit the eligible participants at the appointed time; obtain written informed consent; complete the questionnaire survey; and collect blood specimens, NP swabs, and annal swabs. Feces specimens are limited to diarrheal participants and will be self-collected with sterile containers. Investigators will take them in the next day.

During the investigation, investigators will inform the participants of laboratory results via their preferred mode of contact, such as telephone, email, or express delivery. The identified asymptomatic cases in the survey will immediately report to local CDCs and transfer to medical facilities. The primary benefit of the investigation is to prevent the further spread of the virus.

### 2.10. Data Analysis Plan

On completion of the investigation, data will be imported into the data analysis software (SPSS version 23.0 and SAS version 9.4) for data cleaning and statistical analysis. The prevalences of SARS-CoV-2-specific antibodies in serum and those of SARS-Cov-2, and other respiratory pathogens in NP specimens will be presented with their 95% confidence intervals (CIs). Participants’ characteristics will be described as means ± SDs for normally distributed variables, as medians, and interquartile ranges (IQRs) for non-normally distributed variables, and as frequencies and proportions for categorical variables. Bivariate and multivariable analyses will be performed to identify potential factors associated with infection of SARS-Cov-2 and other respiratory pathogens among study participants. Differences between groups will be compared with independent-samples t tests or Mann–Whitney U tests (for continuous variables), χ^2^ tests or Fisher’s exact tests (for categorical variables), and analysis of variance or Kruskal–Wallis where applicable. Appropriate statistical models (logistic regression models and generalized linear mixed models) will be performed to estimate the odds ratios (ORs) of factors associated with SARS-Cov-2 infection. Adjusted odds ratios (aORs) will be obtained using a multivariable model, including the following covariates: age, gender, occupation, education. Further analysis will be determined upon more discussion. 

A *P*-value <0.05 will be considered statistically significant.

## Figures and Tables

**Table 1:** Probability proportional to size (PPS) sampling of primary sampling units (PSUs) for close contacts.

District	Size of Confirmed Cases	Cumulative Sum of Confirmed Cases	Clusters Sampled
Pudong	60	60	26
Huangpu	6	66	
Xuhui	18	84	83
Changning	13	97	
Jing’an	16	113	
Putuo	11	124	
Hongkou	7	131	
Yangpu	9	140	139
Minhang	19	159	
Baoshan	21	180	
Jiading	9	189	
Jinshan	4	193	
Songjiang	14	207	196
Qingpu	6	213	
Fengxian	9	222	
Chongming	4	226	

Sampling interval (SI) = Total cumulative sum of confirmed cases/Number clusters = 226/4 = 56.5. Seed for random start (RS) is “2020”, RS = 26. Series numbers: 26 (RS), 83 (RS + SI), 139 (RS + 2SI), 196 (RS + 3SI).

**Table 2:** PPS sampling of PSU for floating population.

District	Size of Floating Population (Thousand)	Cumulative Sum of Floating Population (Thousand)	Clusters Sampled (Thousand)	Individuals per District
Pudong	2350.90	2350.90	1125.44	863
Huangpu	166.30	2517.20		
Xuhui	269.80	2787.00		
Changning	174.80	2961.80		
Jing’an	266.80	3228.60		
Putuo	338.00	3566.60	3557.13	863
Hongkou	153.70	3720.30		
Yangpu	268.60	3988.90		
Minhang	1245.90	5234.80		
Baoshan	834.70	6069.50	5988.83	863
Jiading	899.00	6968.50		
Jinshan	270.00	7238.50		
Songjiang	1059.51	8298.01		
Qingpu	707.50	9005.51	8420.53	863
Fengxian	579.41	9584.93		
Chongming	141.87	9726.79		

Sampling interval (SI) = Total cumulative sum of floating population/Number clusters = 9726.79/4 = 2431.70. Seed for random start (RS) is “2020”, RS =1125.44. Series numbers: 1125.44 (RS), 3557.13 (RS + SI), 5988.83 (RS + 2SI), 8420.53 (RS + 3SI). Individuals per district = Expected sample size/Number clusters.

**Table 3:** PPS sampling of at the first stage for school children.

District	Size of School	Cumulative Sum of School	Clusters Sampled	Individuals per District
Pudong	395	395	220	1960
Huangpu	76	471		
Xuhui	98	569		
Changning	59	628		
Jing’an	115	743	696	1960
Putuo	105	848		
Hongkou	80	928		
Yangpu	107	1035		
Minhang	171	1206	1172	1960
Baoshan	170	1376		
Jiading	103	1479		
Jinshan	70	1549		
Songjiang	112	1661	1648	1960
Qingpu	71	1732		
Fengxian	97	1829		
Chongming	74	1903		

Sampling interval (SI) = Total cumulative sum of schools/Number clusters = 1903/4 = 476. Seed for random start (RS) is 2020, RS = 220. Series numbers: 220 (RS), 696 (RS + SI), 1172 (RS + 2SI), 1648 (RS + 3SI). Individuals per district = Expected sample size/Number clusters.

**Table 4:** Respiratory pathogens detection list.

Pathogen	Pathogen	Pathogen
*Adenovirus*	*Influenza A virus H3 (Flu-A_H3)*	*Rhinovirus (A, B, C)*
*Bocavirus*	*Influenza A virus_H1-2009 (Flu-A_H1-2009)*	*Streptococcus pyogenes*
*Cytomegalovirus*	*Influenza B virus* (Flu-B)	*Streptococcus pneumoniae*
*Coronavirus_229E*	*MERS-CoV*	*Bordetella*_pan
*Coronavirus_NL63*	*Human Metapneumovirus 1 (hMPV-1)*	*Bordetella pertussis*
*Coronavirus_OC43*	*Human Metapneumovirus 2 (hMPV-2)*	*Bordetella holmesii*
*Coronavirus_HKU-1*	*Varicella-zoster virus -2*	*Chlamydia pneumoniae*
Enterovirus	*Parechovirus*	*Coxiella burnetii*
*Epstein-Barr virus (EBV)*	*ParaInfuenza-1*	*Legionella pneumophila*
*Herpes Simplex virus 1 (HSV-1)*	*ParaInfuenza-2*	*Mycoplasma pneumoniae*
*Herpes Simplex virus 2 (HSV-2)*	*ParaInfuenza-3*	*Moraxella catarrhalis*
*Human herpesvirus 6 (HHV-6)*	*ParaInfuenza-4*	Mumps
*SARS-CoV*	*Respiratory Syncytial Viral A (RSV-A)*	*Measles virus*
*Influenza A virus (Flu-A)*	*Respiratory Syncytial Viral B (RSV-B)*	
